# 

**Published:** 1875-01-15

**Authors:** 


					﻿No. II.
JANUARY 13, 1873.
Vol. XVI.
THE
Midical IiImmib:

Uemi-ilonthb Journal of flwical minim.
EDITED BY
N. S. DAVIS, M. D., and F. H. DAVIS, M. D.
Subscription Price, Three Dollars per Annum, in advai^e.
Single Numbers, Fifteen Cents.
CHICAGO,
l-LBLISILED AT 65 TC. HANTIOT.I'H STREET.
				

